# PCV2 Triggers PK-15 Cell Apoptosis Through the PLC–IP3R–Ca^2+^ Signaling Pathway

**DOI:** 10.3389/fmicb.2021.674907

**Published:** 2021-06-15

**Authors:** Shuo Wang, Chen Li, Panpan Sun, Jianli Shi, Xiaoyan Wu, Chang Liu, Zhe Peng, Hong Han, Shaojian Xu, Ying Yang, Yao Tian, Jiaxin Li, Hongbin He, Jun Li, Zhao Wang

**Affiliations:** ^1^Shandong Provincial Key Laboratory of Animal Disease Control and Breeding, Institute of Animal Science and Veterinary Medicine Shandong Academy of Agricultural Sciences, Jinan, China; ^2^College of Life Sciences, Shandong Normal University, Jinan, China; ^3^Qingdao Agricultural University, Qingdao, China; ^4^China Institute of Veterinary Drug Control, Beijing, China

**Keywords:** apoptosis, porcine circovirus 2, PLC, IP3R, Cacpsdummy2+, endoplasmic reticulum stress

## Abstract

The endoplasmic reticulum (ER) plays an essential role in Ca^2+^ concentration balance and protein biosynthesis. During infection, the virus needs to complete its life process with the help of ER. At the same time, ER also produces ER stress (ERS), which induces apoptosis to resist virus infection. Our study explored the Ca^2+^ concentration, ERS, and the apoptosis mechanism after porcine circovirus 2 (PCV2) infection. We show here that PCV2 infection induces the increased cytoplasmic Ca^2+^ level and PK-15 cell ER swelling. The colocalization of phospholipase C (PLC) and inositol 1,4,5-trisphosphate receptor (IP3R) in the cytoplasm was observed by laser confocal microscopy. Western blot and quantitative polymerase chain reaction experiments confirmed that PLC and IP3R expression levels increased after PCV2 infection, and Ca^2+^ concentration in the cytoplasm increased after virus infection. These results suggest that PCV2 infection triggers ERS of PK-15 cells *via* the PLC–IP3R–Ca^2+^ signaling pathway to promote the release of intracellular Ca^2+^ and led to cell apoptosis.

## Introduction

Porcine circovirus 2 (PCV2) can cause many diseases in the pig herd, including porcine dermatitis and nephrotic syndrome, A2 congenital tremor (A2CT), porcine proliferative and necrotizing pneumonia, and postweaning multisystemic wasting syndrome, etc. (Magar et al., 2000; Thomson et al., 2001; Dupont et al., 2008). High levels of PCV2 viremia and viral load in tissues, granulomatous inflammation, and immunosuppression were considered the symbols of severe PCV2 infection. To date, the exact pathogenic mechanisms of PCV diseases and PCV-associated diseases (PCVD/PCVAD) are currently unknown. However, many studies have reported the coinfection between PCV2 and other swine pathogens, such as *Haemophilus parasuis*, *Mycoplasma pneumoniae*, and porcine parvovirus, important cofactors that may enhance PCV2 infection and the severity of PCVD/PCVAD (Darwich et al., 2002, 2004; Nielsen et al., 2003).

Previous studies found that the activity of phospholipase C (PLC) could increase the concentration of free Ca^2+^, thus activating the apoptotic signaling pathway (Malli et al., 2007). PLC is an enzyme located in the nuclear membrane, and extracellular stimulated receptors promote PLC activation, hydrolysis of 1,4, 5-triphosphate (IP3), and diacylglycerol and IP3 and releases Ca^2+^ from intracellular stores. Then, the release of Ca^2+^ through inositol 1,4,5-trisphosphate receptor (IP3R) leads to Ca^2+^ depletion in the endoplasmic reticulum (ER) and increases the concentration of Ca^2+^ in the cytoplasm. ER is the main intracellular storage site for Ca^2+^, which has the powerful ability to uptake and release Ca^2+^. The IP3R was an IP3-binding protein and was a Ca^2+^ channel localized on the ER (Zhou et al., 2009; Zhu et al., 2018). Diverse cellular stresses, such as lack of cellular nutrients, hypoxia, acid–base imbalance, or reactive oxygen species accumulation, cause unfolded or misfolded proteins to accumulation inside the ER lumen, a condition known as ER stress (ERS) (Mou et al., 2020), and activate cell damage (Wei et al., 2020). As an adaptive action, the unfolded protein response (UPR) is triggered to decrease the ER protein load (Zhou et al., 2017). When cells cannot recover from ERS, UPR will terminate this adaptive response and trigger cell apoptosis. Many viruses can cause cell apoptosis when infecting host cells. The virus can accelerate or induce apoptosis to promote the release and spread of virions (Galluzzi et al., 2010).

In this study, we aimed to explore the interplay between PCV2 and host cell apoptosis signaling pathways. And we found that PCV2 infection triggers ERS of PK-15 cells by activating the PLC–IP3R–Ca^2+^ signaling pathway to promote the release of intracellular Ca^2+^ and induce cell apoptosis. By exploring the relationship between viruses and apoptosis, it is of great significance to further explore and reveal the pathogenic mechanism of the virus and to provide a new theoretical basis for finding antiviral targets of host cells.

## Materials and Methods

### Cells and Viruses

Cell line PK-15 free of PCV1 contamination was cultured at 37°C in 5% CO_2_ in Dulbecco modified eagle medium (Gibco) supplemented with 8% fetal bovine serum (BIOIND, 04-001-1ACS).

Porcine circovirus 2 virus strain used in this study was stored in Shandong Provincial Key Laboratory of Animal Disease Control and Breeding, Institute of Animal Science and Veterinary Medicine Shandong Academy of Agricultural Sciences.

### Viral Infection and Drug Treatment

PK-15 cells were infected with PCV2 at a multiplicity of infection (MOI, plaque-forming units/cell) of 1. After 72 h, the virus was detected according to the indirect immunofluorescence assay procedure, which proved that the virus had increased in the cells. When performing inhibitor effects experiments, adding 2-APB (Sigma–Aldrich, 100065) and 10 mM 4-PBA (Sigma-Aldrich, Y0000808), a final concentration of 40 μM configured with dimethyl sulfoxide (Sigma-Aldrich, D2650) and 50 μM U73122 (Sigma-Aldrich, U6756) fresh medium was cultured in an incubator.

### Determination of Cytoplasmic Ca^2+^ Concentration in PK-15 Cells

The control group, virus infection group, and inhibitor treatment group were set up. Fluo-4AM kit (Invitrogen, F-14201) and 5 μM Fluo-4AM working solution needed to take 4 μL of 2 mM stock solution and diluted with phosphate-buffered saline (PBS); 50 μL 5 μM Fluo-4AM working solution was added to each well. The plate was placed in the incubator for 30 min, and the fluorescence signal intensity was measured with a fluorescence microplate reader with 490-nm excitation light and 520-nm emission light.

### Apoptosis Detection

The samples were collected 24, 48, and 72 h after the virus infection. The TUNEL assay kit (One Step TUNEL Apoptosis Assay Kit, Beyotime, C1086) was used to visualize apoptotic cells following the manufacturer’s instruction.

### Cell Transmission Electron Microscopy Technique

After the cells were cultured for 24 h, the floating dead cells were washed away with PBS, and the cells were collected in a 1.5-mL centrifuge tube, centrifuged at 3,000*g* for 5 min (the electron microscope fixative was added), fixed at room temperature for 1 h, and observed through a transmission electron microscope.

### Western Blot

Western blot was performed as described previously (Kurien and Scofield, 2006). The membranes were blocked for 1 h and then probed for 1 h with the following primary antibodies: β-actin (Zhongshan Jinqiao TA-09), PCV2 Cap protein monoclonal antibody, anti-rabbit immunoglobulin G, horseradish peroxidase–linked antibody (GeneTex), anti-IP3 receptor antibody (Abcam, ab5804), and anti-PLC beta1/PLC antibody (Abcam, ab233157). The membrane was washed again and then incubated for another hour with the goat anti-mouse secondary antibody (Alexa Fluor^®^ 488) diluted to 1:5,000. ImageJ software was used to perform the gray-scale analysis of western blot bands; the ratio of target protein molecules (IP3R, PLC, and PCV2 Cap) to β-actin was calculated, and the blank ratio of 1.0 was set. Then, the other groups were compared to the control group.

### Real-Time Fluorescence Quantitative Polymerase Chain Reaction of PLC and IP3R

For total RNA extracted samples, retroviruses from Aidlab Company Kit (TURE script 1st Stand cDNA short Kit) were used for reverse transcription operations, respectively, to join primers PLC-F: AGAACGCTGGCTGTGGCTACG, PLC-R: GGAGGGTCTGGGCTTTGGAAGG, IP3R-F:CTTGTGGGCT ACGTGTCTGG, IP3R-R:CTCTTGGGTGTCTTCTTCTCTGG.

AceQ^®^ quantitative polymerase chain reaction (qPCR) SYBR Green Master Mix (Vazyme, Q121) was used for the qPCR experiment.

### Statistical Analysis

Statistical significance was calculated using multiple comparisons in GraphPad Prism software (ns represents no significant difference, *P* > 0.05; ^∗^ represents difference (0.01 < *P* < 0.05); ^∗∗^ represents very significant difference, *P* < 0.01).

## Results

### PCV2 Infection of PK-15 Cells Results in Up-Regulation of Ca^2+^ Concentration

**FIGURE 1:**
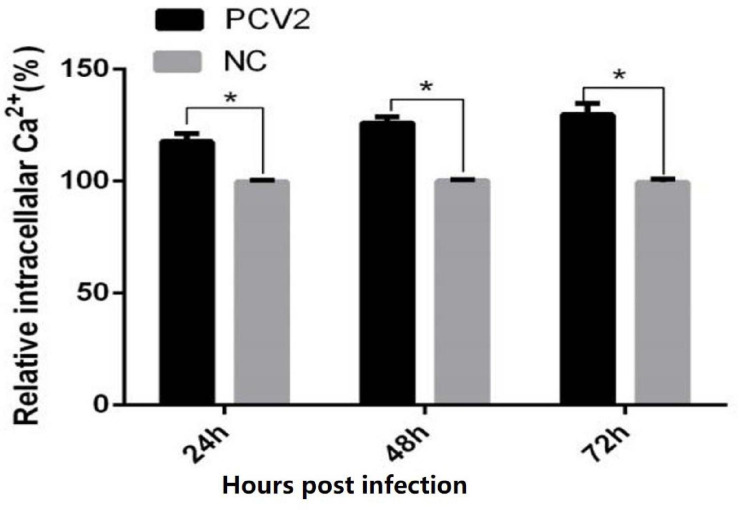
Results of intracellular calcium concentration in cells. When the cells were infected with PCV2 for 72 h, the intracellular Ca^2+^ concentration was measured every 24 h. The figure shows that the Ca^2+^ concentration in PK-15 cells is higher than that in the control group. The data have significant differences after statistical analysis (*P* < 0.05). “*” represents difference (0.01 < *p* < 0.05).

The results of the luciferase marker showed that the levels of cytoplasm-free Ca^2+^ at 24, 48, and 72 h after PCV2 infection were significantly higher in virus-infected cells, compared with uninfected controls, suggesting that PCV2 infection induced the up-regulation of cytoplasm-free Ca^2+^ (Figure 1) (24 h: 17.5 vs. 100%, 0.01 < *P* < 0.05; 48 h: 119.6 vs. 100%, 0.01 < *P* < 0.05; 72 h: 25.7 vs. 100%, 0.01 < *P* < 0.05).

### PCV2 Infection–Induced Apoptosis and ER Swelling

**FIGURE 2:**
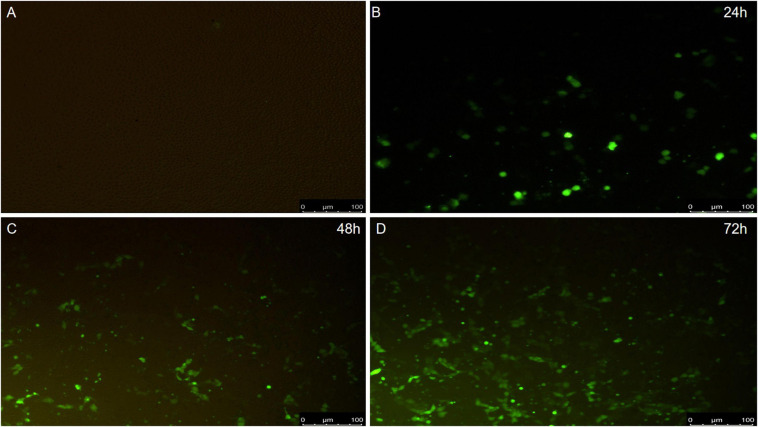
The results of apoptosis. **(A)** Negative control. **(B)** Virus infection group 24 h. **(C)** Virus infection group 48 h. **(D)** Virus infection group 72 h.

To detect whether PCV2 could induce apoptosis, PK-15 cells were infected with the PCV2 2W3 strain. The results showed that PCV2 infection led to increased numbers of apoptosis cells at an indicated MOI at 24 h postinfection (hpi), up to 72 hpi (Figure 2), suggesting that PCV2 infection induced cell apoptosis.

**FIGURE 3:**
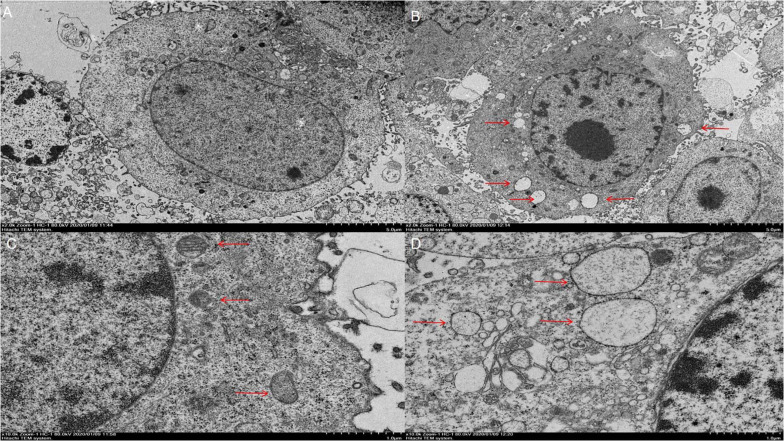
Results of cell electron microscopy. **(A)** Control cells at 5,000× magnification. **(B)** Challenge group cells amplified at 5,000×. **(C)** Control cells at 10,000× magnification, with the arrow pointing to the endoplasmic reticulum. **(D)** Cells in the challenge group at 10,000× magnification. The arrow indicates the endoplasmic reticulum with abnormal enlargement.

We observed that there were significant differences in morphology between the virus-infected cells and uninfected controls. In the virus-infected group, the cytoplasm becomes thinner, the ER is abnormally swollen (Figure 3), the attached ribosomes are reduced, and the perinuclear space is swollen, not found in the uninfected controls. These results demonstrated that PCV2 could cause abnormal swelling of ER structure and morphological changes of PK-15 cells, leading to the phenomenon of ERS. Taken together, these results indicate that PCV2 infection opened the calcium channels in the ER, and Ca^2+^ is released into the cytoplasm, leading to an increase in the concentration of free Ca^2+^ in the cytoplasm, which in turn triggers apoptotic pathways to induce cell apoptosis.

### Colocalization of PLC Protein and IP3R Protein in Cells

**FIGURE 4:**
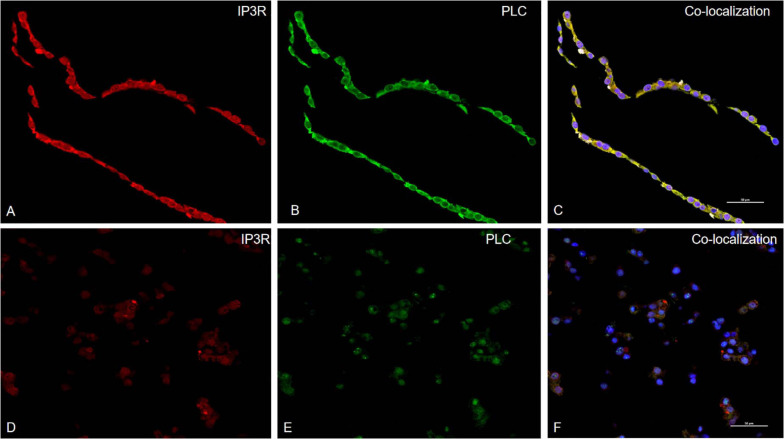
PLC colocalizes with IP3R in PCV2 infect PK-15 cells. Laser confocal immunofluorescence was used to detect the expressions of PLC and IP3R in PK-15 cells infected with PCV2. The red is IP3R protein, the green is PLC protein, and the yellow part is the colocation of the two proteins. **(A–C)** are the expression and colocalization of protein in cells after virus infection. **(D–F)** are the colocalization of the protein in the control group of PK-15 cells without PCV2 infection.

Phospholipase C is a critical signaling enzyme that hydrolyzes PIP2 to generate IP3, which binds to IP3R and stimulates increases in intracellular Ca^2+^. To determine the relationship between PLC and IP3R in PK-15 cells, PCV2 was used to infect PK-15 cells and observed the positional relationship of two proteins in the cell with a confocal laser scanning microscope: the IP3R protein was labeled as red, the PLC protein was labeled as green, and their colocation area was labeled as yellow when PCV2-infected PK-15 cells, PLC, and IP3R colocated in the cells, in the control cells, the colocalization of the two proteins on the membrane was not obvious (Figure 4).

### PCV2 Infection With PK-15 Cells Can Activate the PCL–IP3R–Ca^2+^ Signaling Pathway and Induce Cell Apoptosis

**FIGURE 5:**
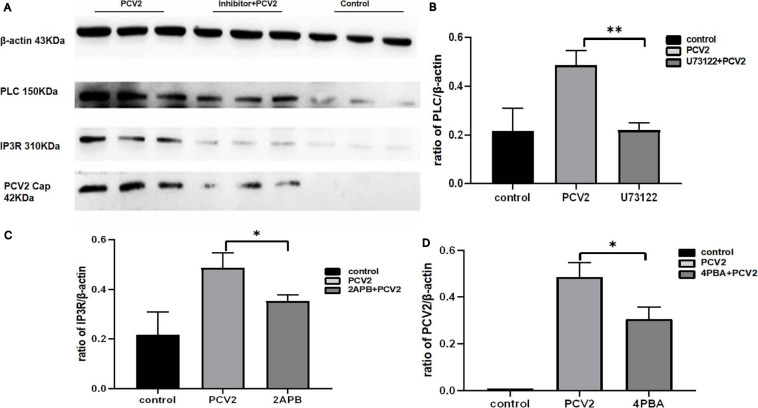
Expression of different proteins after inhibitor addition changes of the control group. **(A)** Western blot results. **(B)** Histogram of PLC protein expression in different experimental groups after the addition of U73122 showed that the PLC expression in the virus infection group with inhibitor was significantly different from the inhibitor group. **(C)** Histogram of IP3R protein expression in different experimental groups after the addition of 2-APB. **(D)** The histogram of PCV2 Cap protein expression in different experimental groups after the addition of 4-PBA showed that the expression of PCV2 Cap protein in host cells significantly decreased after the addition of 4-PBA. “*” represents difference, 0.01 < *p* < 0.05; “**” represents very significant difference, *p* < 0.01.

**FIGURE 6:**
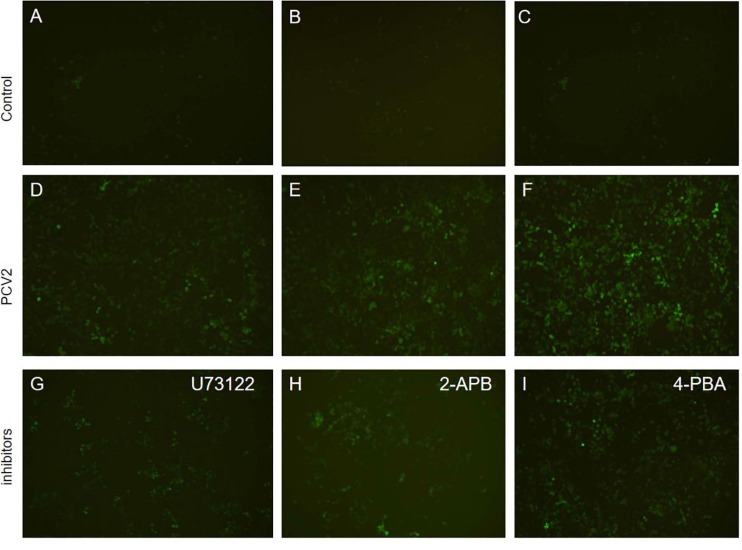
Fluorescence results of apoptosis detection (×200). **(A–C)** Apoptosis in the control group. **(D–F)** Apoptosis of cells infected with PCV2 virus after 72 h. **(G)** Apoptosis after adding PLC inhibitor (U73122). **(H)** Apoptosis after adding IP3R receptor inhibitor 2-APB. **(I)** Apoptosis after adding endoplasmic reticulum stress inhibitor 4-PBA.

**FIGURE 7:**
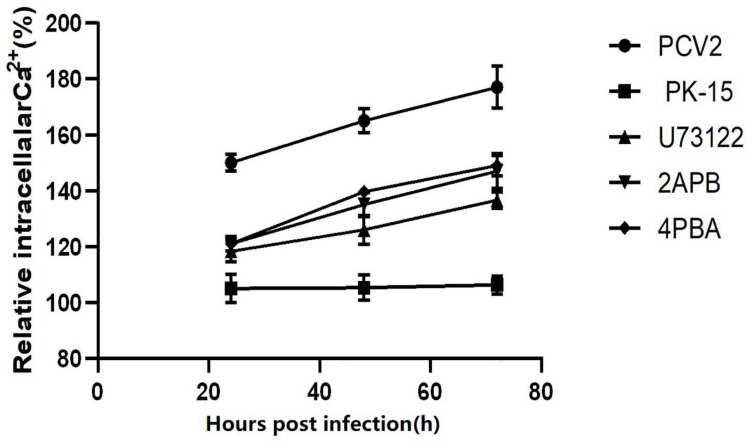
Changes of Ca^2+^ after virus infection. The different marked lines in the figure represent the changes of intracellular Ca^2+^ concentration in different periods of virus infection and PK-15 cells after adding different inhibitors, showing the influence of inhibitors on Ca^2+^ concentration.

**FIGURE 8:**
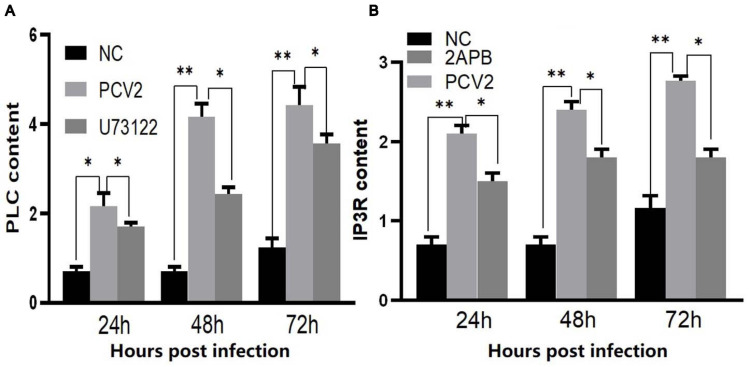
**(A)** Changes in the expression level of PLC mRNA in PK-15 cells during PCV2 infection. **(B)** Changes in the expression level of IP3R mRNA in PK-15 cells during PCV2 infection. “*” represents difference, 0.01 < *p* < 0.05; “**” represents very significant difference, *p* < 0.01.

To prove whether PCV2 induces ERS by activating the PLC–IP3R–Ca^2+^ signaling pathway after infecting PK-15 cells and releases Ca^2+^ into the cytoplasm to promote cell apoptosis. We added ERS reliever 4-PBA, the PLC inhibitor (U73122), and the IP3R inhibitor (2-APB) to the cells and detect apoptosis, Ca^2+^ concentration and protein expression in different experimental groups. The results showed that after the ERS reliever 4-PBA was added into the PCV2 infected cells, resulting in a decrease in the expression of PCV2 Cap protein (Figure 5), and the concentration of Ca^2+^ was significantly lower with the 4-PBA (Figure 6), suggesting that the 4-PBA affected the opening of Ca^2+^ channels on the ER, thereby inhibiting the flow of Ca^2+^ to the cytoplasm, and finally alleviated the apoptosis of PK-15 cells (Figure 7). Besides, after PCV2 infection, the mRNA expression of IP3R and PLC was significantly up-regulated compared with the uninfected group by 3.72- and 2.83-fold (Figure 8).

Furthermore, after adding PLC and IP3R inhibitors, the mRNA expression of IP3R and that of PLC were decreased (Figure 8). The PK-15 cells exhibit an apparent apoptosis disadvantage (Figure 7). The concentration of Ca^2+^ increased with the prolongation of the virus infection time. Still, it was significantly lower than that of virus-infected cells without inhibitor treatment (Figure 6), suggesting that the concentration of Ca^2+^ correlated with the expression of PLC and IP3R in the cell. Under normal circumstances, PLC exists on the cell membrane of PK-15. When cells are stimulated by external factors (such as viral infection), the content of PLC and IP3R in the cytoplasm will increase. Therefore, in western blot results, after infection of cells with PCV2, the PLC and IP3R expression in the cytoplasm increased. At the same time, we added inhibitors of PLC and IP3R to observe the changes in cell protein expression and Ca^2+^ concentration after challenge and found that inhibitors can inhibit protein expression. After adding the ERS relief agent, the content of PCV2 Cap protein in the cell was detected. The inhibitor can alleviate the ERS state of the cell and affect virus replication, so the expression of PCV2 Cap protein is reduced.

In a word, the experimental results indicate to a certain extent that changes in protein expression affect the PLC–IP3R–Ca^2+^ signaling pathway, reducing the intracellular calcium ion concentration, thereby affecting the proliferation and replication of the virus in the cell. These results showed that PCV2 could induce ERS and cause cell apoptosis through the PLC-IP3R signaling pathway to promote viral replication.

## Discussion

As a DNA virus, PCV2 cannot complete the entire life cycle independently, and it must rely on the life cycle of host cells to complete its proliferation. Studies have shown that viral infection could induce apoptosis in cultured PK-15 cells by requiring the activation of caspase-8 and effector caspase-3 pathways (Liu et al., 2005). In addition, compared with the wild-type strain, the pathogenicity of PCV2 deficiency of ORF3 in pigs was reduced (Juhan et al., 2010). Studies have shown that PCV2 is related to lymphocytosis and histopathological infiltration in histopathology and causes apoptosis in mouse and pig models. Apoptosis is closely related to the changes and flow of Ca^2+^ in the ER and mitochondria (Dvorak et al., 2013; Lv et al., 2014). At present, there are few studies on the mechanism of PCV2-inducing apoptosis and the kinetics of Ca^2+^ in PK-15 cells. Therefore, studying the relationship between viral infection and apoptosis is of great significance to further explore and reveal the viral pathogenic mechanism.

After the virus enters the host cell, it replicates itself with the host’s help and threat (Hao et al., 2019). Viruses seek advantages, avoid disadvantages, and also affect the internal environment of host cells. It has been shown that increased Ca^2+^ in the cytoplasm results from the release of ER (Bano and Nicotera, 2007). Current research generally believes that Ca^2+^ is the initial signal of the apoptosis pathway and that high intracellular calcium can trigger cell apoptosis. Early studies showed that PCV2 infection could lead to ERS. In this study, we observed the ER swelling of PK-15 cells after viral infection by transmission electron microscopy and found that PCV2 infection triggered ERS of PK-15 cells through the PLC–IP3R–Ca^2+^ signaling pathway and cause apoptosis.

When cells were stimulated by the external environment, the ES released Ca^2+^ into the cytoplasm. When the concentration of Ca^2+^ reached a certain threshold and discontinuously decreased, the calcium-dependent endogenous endonuclease was activated, and the DNA is decomposed into 180- to 200-bp oligos, leading to apoptosis (Xiang et al., 2017). Ca^2+^ acts as a messenger molecule in cell proliferation, the transmission of external molecular stimuli, and antiviral invasion molecules’ activation (Zhou et al., 2009). Current studies have shown that ER Ca^2+^ homeostasis affects ERS and induces apoptosis through activation of autophagy and inflammation groups (Mou et al., 2020). Virus infection can interfere with ER homeostasis and cause ERS.

To cope with the harmful effects of virus-induced ERS, cells activate key signal transduction pathways, including UPR and intrinsic mitochondrial cell apoptosis. Up to now, approximately 36 viruses have been found to trigger ERS and differentially activate ERS-related signaling pathways (Benali-Furet et al., 2005; Li et al., 2015). Our research found that the apoptosis caused by PCV2 infection of PK-15 cells is related to ERS caused by the increase in intracellular Ca^2+^ concentration. 4-PBA can inhibit cells from entering the S phase. PCV2 replication requires S and G2/M phases. When the cell cycle is blocked, PCV2 cannot replicate (Tung et al., 2015; Zeng et al., 2017). We verified that 4-PBA could alleviate the stress of PCV2 on the ER of PK-15 cells and the expression of PCV2 Cap protein. The use of drugs to interfere with ERS caused by the virus can reduce virus replication and alleviate cell apoptosis.

Inositol 1,4,5-trisphosphate receptor (IP3R) and PLC are Ca^2+^-dependent cellular proteins. PLC stimulates IP3 to bind to IP3R, opens Ca^2+^ channels in ER, and makes Ca^2+^ flow into the cytoplasm. This finding confirmed that PCV2 attacks host cells by affecting the dynamics of intracellular Ca^2+^. As mentioned in previous reports, Ca^2+^ is a limiting cytokine for virus budding. Increasing the concentration of Ca^2+^ in the cytoplasm can enhance virus release (Aliyu et al., 2019).

In this study, we first observed that the colocalization of PLC and IP3R in the cytoplasm of PK-15, which preliminarily verified that the PLC-IP3R pathway in PK-15 cells regulates the flow of Ca^2+^, leads to the occurrence of cell apoptosis. In future research, we can explore the relationship between the PLC–IP3R–Ca^2+^ pathway and the cytokines involved in cell apoptosis, Research in this field will help reveal virus replication and pathogenesis and provide new ideas for future research and antiviral drugs.

## Data Availability Statement

The raw data supporting the conclusions of this article will be made available by the authors, without undue reservation.

## Author Contributions

JL and PS conceived the project. PS and SW designed the experiments. XW, JS, ZP, CaL, HH, and HBH performed most of the experiments. CeL, YT, JXL, YY, and SX contributed to materials and participated in discussion. SW wrote the manuscript. JL and ZW supervised the work and edited the final version of the manuscript which was read and approved by all authors.

## Conflict of Interest

The authors declare that the research was conducted in the absence of any commercial or financial relationships that could be construed as a potential conflict of interest.
